# Engineering the Link: From Genome Interaction Maps to Functional Insight

**DOI:** 10.1002/adbi.202500525

**Published:** 2025-11-12

**Authors:** Frido Petersen, Simon Westermann, Valeriia Smialkovska, Jan Mathony, Angelika Feldmann, Dominik Niopek

**Affiliations:** ^1^ Pharmaceutical Biology Institute of Pharmacy and Molecular Biotechnology Faculty of Engineering Sciences Heidelberg University 69120 Heidelberg Germany; ^2^ Mechanisms of Genome Control German Cancer Research Center (DKFZ) Im Neuenheimer Feld 581 69120 Heidelberg Germany; ^3^ Faculty of Bioscience Ruprecht‐Karls‐University of Heidelberg 69120 Heidelberg Germany

**Keywords:** 3D genome organization, chromatin looping, CRISPR, gene regulation, genome engineering, information processing

## Abstract

The 3D organization of the genome constitutes a spatial layer of information processing that helps govern gene expression and thus cell function. Advances in chromosome conformation capture sequencing have enabled detailed assessment of chromatin architecture, from enhancer–promoter loops to topological domains and higher‐order contacts, across cell types and developmental states. While the ability to investigate genome conformation is maturing, the field faces a central challenge: The link between chromatin interactions and cellular function remains largely correlative, leaving their causality unresolved. This review explores how recent developments in genome engineering enable the targeted manipulation of 3D chromatin architecture – specifically DNA loops – to illuminate causal links between genome structure and function. Synthetic strategies are introduced that rewire enhancer–promoter communication through engineered chromatin loops, leveraging programmable DNA‐binding platforms such as zinc fingers, transcription activator‐like effectors (TALEs), and CRISPR‐Cas9. The current limitations of these approaches related to efficiency, scalability, and specificity are also highlighted, and the strategies to address them are outlined. As these systems mature, programmable 3D genome engineering is emerging as a transformative pillar of synthetic biology, complementing sequence‐based editing as a core modality for both understanding and ultimately reprogramming genome function.

## Graphical abstract

Advances in chromosome conformation capture have revealed the genome's 3D organization, yet its causal impact on gene regulation remains elusive. This review highlights emerging genome‐engineering tools ‐ zinc fingers, TALEs, and CRISPR‐Cas9 ‐ that enable targeted manipulation of chromatin loops to dissect structure–function relationships. It discusses current limitations and future opportunities for programmable 3D genome engineering in synthetic biology.

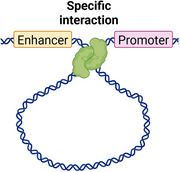

## 1 Introduction

Over the past decade, genome engineering has undergone a revolution. Naturally‐derived CRISPR‐Cas effectors and their engineered derivatives, including base and prime editors, now allow DNA sequences to be modified with remarkable precision, efficiency, and flexibility across the tree of life.^[^ ^1^, ^2^, ^3^, ^4^, ^5^, ^6^, ^7^ ^]^ As a result, sequence‐level manipulation of the genome has become a standard procedure effectively applied by labs around the globe. Although the linear DNA sequence provides the foundation of genetic information, advances in genome mapping and epigenetic editing have made it clear that gene regulation, and thus cell function and organismal behavior, are only partly defined by sequence alone. In complex organisms, the genome is organized at an additional layer – its 3D topology and relative positioning within the nucleus. A major driver of this spatial architecture is protein‐mediated chromatin interactions that fold chromatin into domains and higher‐order structures. Remarkably, this spatial DNA organization is highly dynamic,^[^ ^8^ ^]^ cell‐type specific^[^ ^9^, ^10^ ^]^ and is linked to diverse cellular processes, including transcription,^[^ ^11^ ^]^ DNA repair,^[^ ^12^ ^]^ and ultimately health and disease.^[^ ^13^, ^14^ ^]^

**Figure 1:**
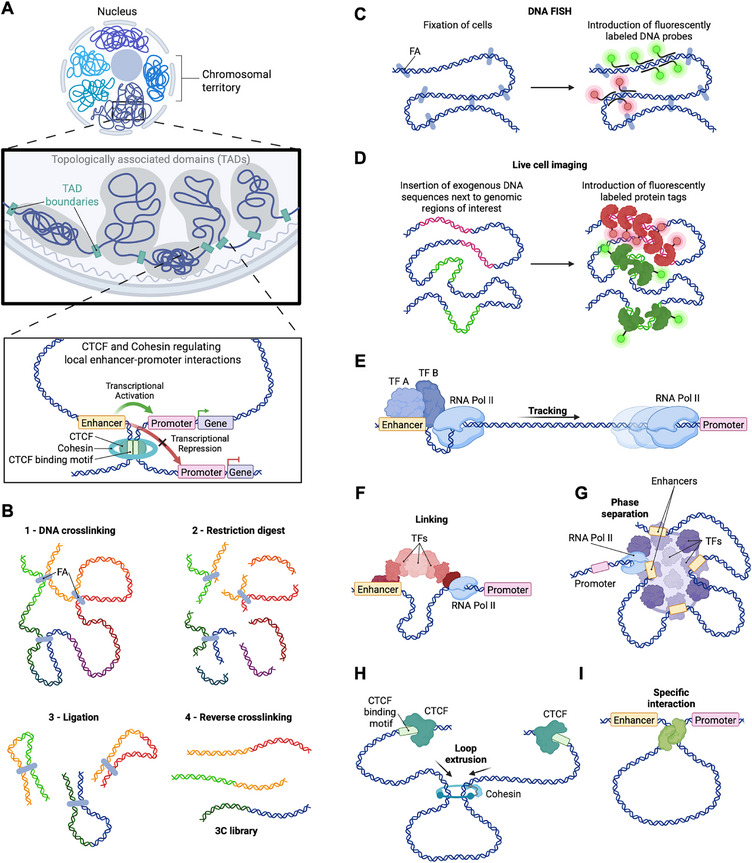
Methods to analyze 3D genome organization and proposed models of enhancer–promoter communication. A) Structural hierarchy of the genome. Inside the nucleus, chromosomes occupy distinct territories subdivided into topologically associating domains (TADs). Within and between these domains, long‐range, dynamic DNA contacts mediate promoter‐enhancer communication. B) Chromosome conformation capture methods. Formaldehyde (FA) cross‐linking and restriction digestion capture spatially proximal DNA fragments. Fragments are then proximity‐ligated to form chimeric ligation products, purified and analyzed (typically by paired‐end sequencing). C) DNA FISH. Cells are fixed with FA and permeabilized. They are then hybridized with fluorescent probes targeting selected genomic loci, thus enabling precise spatial localization. D) Live‐cell imaging. DNA tags are inserted adjacent to genomic regions of interest; labeled DNA‐binding proteins enable visualization of chromatin dynamics in living cells. E) Tracking model. Transcription factors (TFs) and RNA Pol II assemble at the enhancer; RNA Pol II tracks along the DNA toward the promoter. F) Linking model. Multiple TFs assemble into a protein bridge that guides RNA Pol II from enhancer to promoter. G) Phase separation model. Multivalent interactions between TFs, coactivators, and RNA Pol II generate dynamic condensates at enhancers and promoters, concentrating regulatory factors and facilitating communication without direct contacts. H) Loop extrusion model. The ring‐shaped cohesin complex extrudes chromatin until halted by CTCF‐bound sites, thereby forming insulated domains and promoting internal enhancer–promoter interactions. I) Specific interaction model. DNA‐binding proteins mediate physical contacts between distant sites, bringing promoters and enhancers into proximity.

Various mechanisms have been proposed to connect chromatin conformation with gene regulation, covering the range of chromosome territories and megabase‐scale topologically associating domains (TADs) down to local enhancer–promoter interactions (**Figure** **1**A).^[^ ^15^, ^16^, ^17^, ^18^ ^]^ Communication between enhancers and promoters can be explained through several models with the largest amount of evidence supporting a contact dependent communication.^[^ ^19^, ^20^, ^21^, ^22^, ^23^, ^24^, ^25^ ^]^ These models directly link DNA spatial organization to gene regulation and cellular phenotype, showing that the genome processes information both in sequence and in space. The importance of this spatial information processing layer is underscored by the fact that disruptions of 3D genome organization are implicated in diverse diseases. Enhancer miswiring underlies developmental disorders such as polydactyly^[^ ^26^, ^27^ ^]^ and fragile X syndrome.^[^ ^28^ ^]^ Moreover, enhancer hijacking, where oncogenes are aberrantly activated by spatially misplaced regulatory elements that can also form aberrant interactions, is a frequent event in cancer.^[^ ^29^, ^30^, ^31^, ^32^ ^]^ Massive parallel live‐cell imaging measurements in *Drosophila* embryos established the link between transcriptional activation and spatial proximity.^[^ ^33^ ^]^ Still, chromatin looping may not always be associated with transcription. For instance, transcription bursts are uncoupled from promoter–enhancer interactions^[^ ^34^ ^]^ and interactions with regulatory enhancers may be resolved prior to transcriptional activation^[^ ^35^, ^36^, ^37^ ^]^ or pre‐formed several hours ahead of it.^[^ ^38^ ^]^

These findings highlight the widespread nature of spatial genome regulation, yet key questions regarding the functional significance of observed chromatin interactions remain:

To what extent are specific 3D contacts essential for gene regulation? Which interactions are causal, which are redundant, and which are merely correlative?

Addressing these questions requires moving beyond purely descriptive maps toward perturbation‐based experiments using locus‐specific, reversible, and scalable tools that can create, stabilize, or dissolve DNA loops with temporal precision and minimal genetic payload.

In this review, we examine the biology and engineering of chromatin loops: how they are measured, how their functional importance is evaluated, and how synthetic biology is starting to rewire them. Of note, we do not discuss in detail other higher‐order features of chromatin organization, such as chromosome territories or nuclear positioning, and refer readers to the extensive, dedicated literature on these topics.^[^ ^39^, ^40^, ^41^ ^]^ While proof‐of‐principle methods to alter DNA loops already exist, overcoming their limitations in specificity, temporal control, reversibility, multiplexing, and delivery will fully unlock the power of these technologies. The field is moving toward a future in which 3D‐genome engineering complements sequence‐level editing, bringing us closer to a comprehensive understanding – and ultimately programmable control – of both the genetic code and its spatial context.

## 2 Methods to Study Chromatin Interactions

Although physical interactions between distant DNA elements have been proposed to be central to transcription control since the 1980s,^[^ ^16^ ^]^ the rational analysis of chromatin interactions in a genomic context only began with the establishment of Chromosome Conformation Capture (3C).^[^ ^42^ ^]^ 3C uses formaldehyde cross‐linking to preserve proximities, restriction digestion, and proximity ligation, followed by locus‐specific quantification – establishing a foundation for measuring interactions between two defined loci (Figure 1B). This framework expanded to 4C (one‐vs‐all) and 5C (many‐vs‐many), allowing to assess larger number of targets,^[^ ^43^, ^44^ ^]^ and then to Hi‐C, which incorporates biotinylation and high‐throughput sequencing to generate genome‐wide contact maps.^[^ ^45^ ^]^ Numerous derivatives adapt 3C libraries for targeted or higher‐resolution readouts (e.g., Capture‐(Hi‐)C, Micro‐C, HiChIP)^[^ ^46^, ^47^, ^48^, ^49^ ^]^ and multiway contacts (e.g., Tri‐C),^[^ ^50^ ^]^ each providing different throughput and resolution. In general, 3C‐based assays report population‐averaged contact frequencies captured by proximity ligation rather than absolute distances, motivating orthogonal single‐cell spatial measurements.

Complementary imaging approaches directly assess spatial distances and colocalization between selected genomic loci. DNA‐FISH employs fluorescently labeled DNA probes that hybridize to complementary sequences in situ in fixed cells or tissues, enabling precise localization of selected genomic regions and widely used analyses of genome architecture (Figure 1C).^[^ ^51^, ^52^ ^]^

Live‐cell imaging methods typically rely on the insertion of exogenous DNA sequences adjacent to the genomic loci of interest. Fluorescently tagged proteins, expressed either transiently or stably, bind to these sequences and enable visualization of the corresponding genomic regions (Figure 1D). Operator–repressor arrays such as tetO/tetR can mark enhancers and promoters for colocalization analysis.^[^ ^34^, ^53^ ^]^ Fluorescent protein tagging systems such as HaloTag provide covalent, rapid labeling of fusion proteins under physiological conditions and are frequently combined with DNA‐binding modules for robust live imaging.^[^ ^54^ ^]^ ParS/ParB‐derived systems, including ANCHOR, enable single‐locus tracking and have revealed transcription‐initiated local confinement of chromatin domains.^[^ ^55^ ^]^ Recent multicolor implementations extended this strategy to simultaneous two‐locus tracking: mParSpot, for example, uses ParB/ParS and Noc/NBS modules inserted at defined sites to visualize pairs of loci and quantify their distances in living cells, including promoter–terminator dynamics across hundreds of kilobases.^[^ ^56^ ^]^

In sum, 3C‐derived proximity ligation assays map genome‐wide contact propensities at high throughput, whereas imaging, DNA‐FISH in fixed cells and live‐cell locus tagging, provide single‐cell spatial distances and temporal dynamics. Together, they offer orthogonal, independent approaches to assess the 3D organization of the genome.

## 3 Regulation of the 3D Genome Structure

Global and locus‐specific structural analyses revealed that the 3D genome is organized at multiple scales. Initially identified as a “checkerboard” pattern in Hi‐C contact maps, compartments bring together biochemically similar chromatin regions that can be separated by several megabases.^[^ ^45^ ^]^ At the next level, megabase‐sized TADs were first characterized in mammalian cells in 2012 by applying 5C and Hi‐C.^[^ ^17^, ^18^ ^]^ At even finer resolution, local features such as chromatin loops and stripes have been observed.^[^ ^57^ ^]^ These nested structures, shaped by numerous DNA‐binding and architectural factors, form the conformational framework for the communication between gene regulatory regions.

### 3.1 Models of Enhancer–Promoter Association

Several physical models have been proposed to explain how enhancers communicate with distant promoters in the context of observed 3D genome organization. In the *tracking model*,^[^ ^58^ ^]^ transcriptional machinery or associated factors load at an enhancer and move along the intervening chromatin toward the promoter (Figure 1E). In support of this model, the insertion of an insulator between the epsilon‐globin and its enhancer located 10 kb away, reduced gene transcription.^[^ ^59^ ^]^ However, it was shown that enhancers can skip the nearest promoter,^[^ ^60^, ^61^ ^]^ implying the existence of other mechanisms of longer‐range enhancer‐promoter communication.

The *linking model* proposes that chromatin‐bound factors assemble a contiguous protein scaffold that connects enhancer and promoter without requiring direct looping (Figure 1F).^[^ ^19^ ^]^ In an intriguing variation of this model, small loops are formed within the intervening chromatin, thereby bringing enhancers and promoters into indirect proximity, again without the need for a direct interaction.^[^ ^58^ ^]^ Nested loops within Micro‐C profiles could result from such linking.^[^ ^62^ ^]^

According to the *phase separation* model, multivalent interactions among transcription factors, coactivators, and RNA polymerase concentrate regulatory components in dynamic condensates at enhancers and promoters, which facilitate communication even in the absence of specific contacts (Figure 1G).^[^ ^63^, ^64^, ^65^ ^]^ While this model is supported by condensate formation around super‐enhancers, it remains challenging to assess its causal effect on transcription in vivo.

*Loop extrusion* relies on ring‐shaped complexes of DNA binding proteins that continuously extrude chromatin loops until halted by boundary elements. Such activity may increase the probability of enhancer–promoter encounters within the same extruded topological domain (Figure 1H). This model provides a mechanism for the emergence of TADs in the genome and is supported by polymer modeling as well as by perturbation studies.^[^ ^20^, ^21^, ^22^ ^]^ However, removing cohesin does not ablate all enhancer‐promoter loops,^[^ ^66^ ^]^ suggesting additional, more specific mechanisms.

Finally, enhancers and promoters could also communicate with each other via the formation of specific targeted *loops*, that are readily visible as punctate interactions in Hi‐C data (Figure 1I) and could result either from direct interactions or tethering.^[^ ^23^, ^24^, ^25^ ^]^

Given the evidence supporting these mechanisms, they are likely not mutually exclusive and instead presumably operate in combination depending on genomic locus, cell state, and signaling context.

### 3.2 Molecular Mechanisms of Contact‐Dependent Models

Loop extrusion has emerged as a key mechanism of genome organization, explaining TADs and topological stripes as a product of the interplay between cohesin and CCCTC‐binding factor (CTCF). Thereby, cohesin, a ring‐shaped multiprotein complex, extrudes chromatin loops until it is stalled at convergently oriented, occupied CTCF motifs.^[^ ^20^, ^21^, ^22^, ^67^ ^]^ They interact dynamically with each other and with DNA, producing multiple transient DNA conformations.^[^ ^68^ ^]^ Importantly, simple CTCF–CTCF bridging by cohesin appears relatively rare (≈8% of alleles for a given loop at any given time) and unstable (10–30 min).^[^ ^8^ ^]^ On the contrary, acute cohesin depletion often yields only modest changes in steady‐state transcription,^[^ ^21^, ^22^, ^69^ ^]^ but leads to more dramatic effects in long‐range transcriptional regulation of gene activation.^[^ ^70^ ^]^ In line with that, cohesin‐dependence of gene expression correlates with chromatin loop length.^[^ ^71^, ^72^ ^]^ Moreover, cohesin loss has been reported to strengthen other higher‐order features, such as compartmental segregation and ultra‐long‐range interactions among super‐enhancers and Polycomb‐bound regions.^[^ ^21^, ^22^, ^69^ ^]^ Thus, cohesin can both generate local connectivity and promote distinct long‐range contacts when absent, a duality that likely underlies its complex, context‐dependent effects on transcription.

A common mechanistic assumption is that TADs delimit the search space for enhancer‐promoter contacts.^[^ ^26^, ^73^ ^]^ Still, several studies detect substantial interactions across TAD boundaries, consistent with TADs being statistical features of population averages rather than hard barriers in single cells.^[^ ^68^, ^74^, ^75^ ^]^ Together with the observation that promoter‐enhancer loops can persist without cohesin,^[^ ^66^ ^]^ this suggests the existence of other molecular mechanisms to support them.

Beyond CTCF/cohesin, Yin Yang 1 (YY1) functions as an architectural factor capable of simultaneously engaging enhancers and promoters,^[^ ^76^ ^]^ whereas the cofactor LIM domain–binding protein 1 (LDB1) promotes chromatin contacts independently of cohesin and CTCF, and was one of the first proteins to be functionally validated as a mediator of enhancer–promoter looping.^[^ ^77^, ^78^ ^]^

Perhaps unsurprisingly, given their relationship with gene activation, several histone modifying complexes,^[^ ^79^, ^80^, ^81^ ^]^ their catalytic activity,^[^ ^82^ ^]^ associated histone modifications^[^ ^79^, ^83^ ^]^ and their readers^[^ ^84^ ^]^ have been proposed to promote interactions between gene regulatory regions. Notable examples include Polycomb repressive complexes (PRC) 1 and 2^[^ ^80^, ^81^ ^]^ and the H3K4me1 methyl transferase (KMT2).^[^ ^79^ ^]^

Many of the proposed looping factors stabilize, recruit or otherwise regulate other factors, and may therefore indirectly contribute to loop formation. Mediator has generally emerged as an important structural component, with both its RNA Pol II‐associated^[^ ^85^ ^]^ and its poised forms^[^ ^35^, ^86^ ^]^ implicated in the regulation of promoter interactions. In addition to transcription co‐factors, elongating RNA Pol II has also been implicated in the regulation of productive interactions.^[^ ^87^, ^88^ ^]^

Alongside the factors that form loops *de novo*, several ligand‐activated receptors were demonstrated to strengthen pre‐existing chromatin interactions. Examples include the estrogen receptor alpha (ERα)^[^ ^89^ ^]^ glucocorticoid receptor^[^ ^90^ ^]^ and androgen receptor.^[^ ^91^ ^]^

Together, these directly and indirectly loop‐associated factors create opportunities for enhancer‐promoter encounters. The variety of involved factors and their interdependence highlights the challenge to disentangle their contribution to transcription versus genome architecture.

### 3.3 Chromatin Loops are Dynamic in Cell‐Type Transitions

DNA‐binding factors that regulate enhancer–promoter interactions bind in a cell‐type‐specific manner leading to genome reorganization during transitions between cell types, when new transcriptional programmes are established. As expected for cell‐type‐specific enhancers, many enhancer–promoter interactions are formed *de novo* during differentiation and are specific to a given cell type.^[^ ^10^, ^23^, ^37^, ^92^ ^]^ Time‐course analysis shows that chromatin contacts may precede transcriptional onset by several hours during *Drosophila* embryogenesis^[^ ^38^ ^]^ and embryonic stem cell differentiation.^[^ ^9^, ^37^, ^81^ ^]^ Moreover, both transcriptional activation and repression involve regulatory contacts that form transiently throughout differentiation^[^ ^37^, ^93^ ^]^ and regulatory enhancers can lose contacts with their cognate promoters prior to activation.^[^ ^35^, ^36^ ^]^ These observations suggest that proximity may not always be required at the time point of active transcription.

Together, the above observations emphasize the temporal pattern of enhancer–promoter interactions, yet the significance of this temporality, as well as the importance of the interactions themselves, remains largely unclear. Therefore, understanding when promoter–enhancer interactions are required is essential, highlighting the need for experimental approaches capable of targeted contact modulation to fully unravel the mechanistic principles governing genome regulation during development.

## 4 Engineering Chromatin Looping

Beyond the compelling evidence that the spatial genome architecture is linked to gene expression, there is no consensus on how exactly enhancers engage promoters and when this engagement governs transcription. Divergent models of enhancer–promoter interactions (Section 3.1) underscore the need for perturbation‐based approaches, actively imposing, disrupting, or multiplexing chromatin contacts, to establish causality and mechanistically link 3D genome architecture to cellular function.

### 4.1 Inducing Chromatin Looping with DNA‐Binding Proteins

Rational re‐engineering of chromatin loops can be achieved with DNA‐binding proteins that are both programmable and capable of high‐affinity, high‐specificity binding to selected genomic loci. Zinc finger proteins (ZFPs) can be engineered to recognize specific DNA triplets via modular α‐helices that contact the major groove^[^ ^94^ ^]^ and can be further assembled in tandem to target longer sequences with increased specificity. Originally developed for genome editing through fusion with FokI nucleases^[^ ^95^ ^]^ ZFPs were successfully explored as a protein‐based platform for chromatin loop engineering.

**Figure 2:**
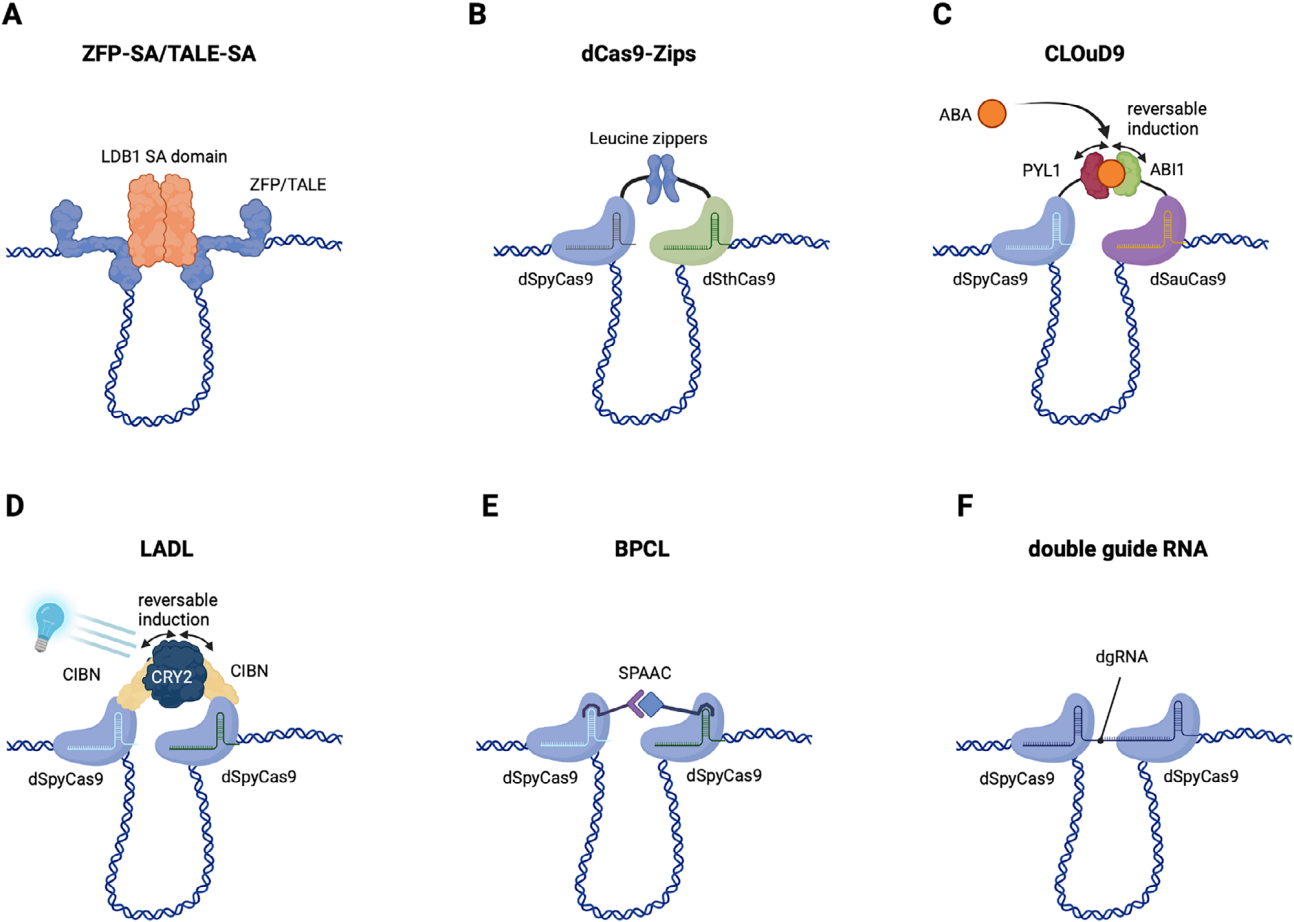
Overview of engineered chromatin‐looping strategies. Cartoons A–F) show the core DNA‐binding modules and interaction mechanisms. A) ZFP–SA or TALE–SA, a zinc‐finger or TALE protein fused to the LDB1 self‐associating (SA) domain to tether distal elements.^[^ ^36^, ^77^ ^]^ B) dCas9‐Zips, paired dCas9 orthologs fused to complementary leucine‐zippers to bridge two loci.^[^ ^115^ ^]^ C) CLOuD9, two distinct dCas9 orthologs fused to the ABA‐responsive PYL1/ABI1 pair for chemically inducible, reversible looping in mammalian cells.^[^ ^118^ ^]^ D) LADL, a single dCas9–CIBN anchor that recruits the photoreceptor CRY2 on blue‐light illumination for rapid, tunable looping.^[^ ^121^ ^]^ E) BPCL, engineered sgRNA scaffolds that recruit complementary, chemically modified oligonucleotide adaptors that are joined in situ by orthogonal click chemistries (SPAAC/SPIEDAC) to produce selective, multiplexable, and photocleavable links.^[^ ^122^ ^]^ F) dgRNA, a covalently linked double‐guide RNA that simultaneously recruits two dSpyCas9 proteins from a single expression cassette for compact, programmable looping (validated in bacteria).^[^ ^123^ ^]^ Colored shapes indicate DNA binders and interaction modules; small icons denote induction modality (if applicable).

Deng et al. pioneered a synthetic looping strategy by fusing ZFPs to the self‐associating (SA) domain of LDB1, a transcriptional cofactor known to promote enhancer‐promoter contacts. The ZFP component of the fusion protein serves as a sequence‐specific tether to the enhancer and promoter region, whereas the SA domain mediates loop formation (**Figure** **2**A).^[^ ^77^ ^]^

In parallel, another class of programmable DNA‐binding proteins, namely transcription activator‐like effectors (TALEs), has been leveraged to manipulate genome topology. Originally derived from plant pathogenic bacteria, TALEs recognize specific DNA sequences through a modular array of repeats, each recognizing a single base via a defined amino acid code, which allows for more predictable and flexible targeting compared to ZFPs.^[^ ^96^ ^]^ Like ZFPs, TALEs have been repurposed as a protein‐only platform for chromatin loop induction. For example, the SA domain was fused to a TALE and targeted to the *Shh* locus (Figure 2A), successfully inducing chromatin loop formation, analogous to previous ZFP‐SA approaches at the *β‐globin* locus.^[^ ^36^, ^77^ ^]^

While both ZFPs and TALEs offer powerful platforms for targeted chromatin modulation, they have important limitations. ZFPs require labor‐intensive design and cloning, and their engineering success often varies. TALEs, by contrast, are more straightforward to program, but their large size and repetitive architecture pose challenges for expression and delivery. Together, these constraints make both platforms difficult to scale for high‐throughput applications. Moreover, multiplexed looping with ZFPs or TALEs, e.g., to create several distinct, simultaneous chromatin contacts, would demand a separately engineered, delivered, and expressed ZFP/TALE variant for each targeted locus, greatly complicating implementation.

Inserting repeat DNA operator arrays at multiple genomic sites has been used as an alternative to programmable DNA‐binding proteins to enforce artificial chromatin loops. In an early study, in 2001, Kostriken and Wedeen inserted large lactose operator (lacO) arrays at paired sites on *S. cerevisiae* chromosome III, near HML (Hidden MAT Left), MAT (the active mating‐type locus), or HMR (Hidden MAT Right).^[^ ^97^ ^]^ They then expressed an engineered, tetrameric lactose repressor (LacI) to act as a protein cross‐bridge, thereby enforcing defined interphase loops. This approach enforced HML–MAT proximity biased donor choice during mating‐type switching toward HML, directly demonstrating that engineered chromosomal loops can causally influence homologous‐recombination outcomes.^[^ ^97^ ^]^ Building on this approach, Nannas & Murray flanked the centromere on yeast chromosome III with lacO arrays to tether sister centromeres, again using tetrameric LacI.^[^ ^98^ ^]^ The tether reduced inter‐kinetochore stretch but did not activate the spindle assembly checkpoint or delay anaphase, leading to the conclusion that the checkpoint senses intra‐kinetochore rather than inter‐kinetochore tension.^[^ ^98^ ^]^ Extending this line of work, Du et al. developed Chemically Induced Chromosomal Interaction (CICI), a rapamycin‐inducible tethering system in budding yeast that installs LacO/TetO arrays at chosen loci and expresses LacI–FKBP12 and TetR–FRB to acutely force contacts.^[^ ^99^ ^]^ They showed that induced interactions can be established across diverse intra‐ and inter‐chromosomal pairs. Interestingly, while looping efficiencies via CICI were broadly similar across different loci pairs, a slower loop forming kinetics was observed for initially distant/low‐contact loci (according to Hi‐C).^[^ ^99^ ^]^

While generally modular, all of the above methods require substantial protein or genomic redesign to specify each new contact. These limitations underscore the need for more versatile, scalable systems to manipulate the 3D genome.

### 4.2 CRISPR‐Based Looping Techniques

The CRISPR‐Cas system (for clustered regularly interspaced short palindromic repeats (CRISPR)/CRISPR‐associated (Cas)), originally discovered as a prokaryotic defense mechanism against bacteriophages^[^ ^100^ ^]^ has become the prime technology for genome editing.^[^ ^1^, ^2^, ^3^ ^]^ At their core, CRISPR systems comprise Cas effector proteins that act as nucleases to target and cleave specific DNA or RNA sequences. Target specificity is provided by an RNA guide that base‐pairs with the complementary DNA target sequence. In the widely used CRISPR–Cas9 system, a class 2, type II CRISPR effector, target recognition is determined by a ≈20‐nucleotide spacer within a single‐guide RNA (sgRNA, an engineered fusion of the natural crRNA and tracrRNA) and requires a protospacer‐adjacent motif (PAM) immediately adjacent to the spacer‐complementary DNA.^[^ ^1^ ^]^ Re‐targeting Cas9 to a different DNA sequence is straightforward and only requires re‐design of the spacer part of the RNA guide. This enables genome editing in high‐throughput for a fraction of the costs compared to TALE or ZFP technologies. Not surprisingly, Cas9‐mediated genome editing has been used extensively to dissect 3D genome architecture and the functional relevance of chromatin loops.^[^ ^26^, ^76^, ^101^, ^102^, ^103^, ^104^, ^105^, ^106^, ^107^, ^108^, ^109^, ^110^, ^111^ ^]^ We will not discuss these studies in detail here, since they mostly rely on conventional CRISPR gene editing (see Section 4.4).

Complementary to nuclease‐based perturbations, catalytically inactive “dead” Cas9 (dCas9) variants, generated by mutating the RuvC and HNH nuclease domains, retain RNA‐guided DNA binding without cleavage, thereby converting Cas9 into an easily programmable DNA binder.^[^ ^112^, ^113^ ^]^ These RNA‐guided scaffolds underpin artificial chromatin‐loop perturbation strategies that alter 3D architecture without changing the DNA sequence. Tarjan et al., for instance, deployed dCas9‐derived epigenetic modifiers to displace CTCF from specific insulators.^[^ ^114^ ^]^ More precisely, they leveraged dCas9 fusion to a Krüppel‐Associated Box (KRAB) domain to induce repressive histone marks or DNA methyltransferases to trigger local DNA methylation, thereby reducing CTCF occupancy at specific CTCF binding motifs and thus altering genome topology.

Apart from perturbing chromatin binding of native proteins involved in orchestrating chromatin folding, such as CTCF, several systems for the directed induction of chromatin looping have been created on the basis of dCas9.

Hao et al. developed dCas9‐Zips, a looping system employing *Streptococcus pyogenes* (d*Spy*Cas9) and *Streptococcus thermophilus* (d*Sth*Cas9) dCas9 orthologs fused to leucine zippers (Figure 2B).^[^ ^115^ ^]^ The idea is that the two different dCas9 orthologs have different PAM and sgRNA requirements, thereby being able to individually program them within the same cell for specific DNA sequences. The leucine zipper, in turn, will tether both dCas9s post‐translation, hence establishing a bivalent protein complex (dCas9‐Zips). Targeting the dCas9‐Zips proteins to distal genomic loci in *Escherichia coli* (*E. coli*) induced looping‐dependent transcriptional repression. The efficiency of this effect was observed to depend on the distance between sites on the genome sequence. Building on earlier observations that large loops in bacterial chromosomes can form via smaller, nested loops^[^ ^116^, ^117^ ^]^ dCas9‐Zips apparently created an inner loop that facilitated outer‐loop formation thereby enhancing gene repression. Subsequent optimization by genetically fusing the two dCas9 orthologs into a single polypeptide eliminated the need for leucine zipper–mediated dimerization, improving the efficacy of this approach. The dCas9‐Zips strategy is conceptually appealing also for use as a tool in mammalian cells which, however, has yet to be demonstrated. Another important consideration regarding the system's use is that expression of dCas9‐Zips will establish static interactions, which prohibit time‐resolved perturbations of chromatin interactions.

Conceptually related work by Morgan et al. overcomes these two limitations.^[^ ^118^ ^]^ The authors developed CLOuD9, a chemically inducible chromatin‐looping system that has been successfully implemented in mammalian cells and is inducible with an exogenous trigger, namely abscisic acid (ABA). CLOuD9 employs dSpyCas9 and *Staphylococcus aureus* dCas9 (dSauCas9), which were fused to PYL1 and ABI1, respectively. PYL1 (Pyrabactin Resistance Like 1) is an ABA receptor; ABI1 (Abscisic Acid Insensitive 1) a type 2C protein phosphatase (PP2C) and negative regulator of ABA signaling. Upon addition of ABA, dimerization between the PYL1 and ABI1 parts is induced^[^ ^119^ ^]^ thereby bringing together the targeted DNA loci and resulting in loop formation (Figure 2C).^[^ ^118^ ^]^ Using this system, the authors induced LCR–β‐globin contacts in both K562 (erythroid, euchromatic) and HEK293T (human embryonic kidney derived) cells and detected increased 3C cross‐linking in both backgrounds. Remarkably, transcriptional activation, increased H3K4me3, and RNA Pol II occupancy were observed only in K562s and not in 293Ts.^[^ ^118^ ^]^ By contrast, CLOuD9‐mediated tethering of the *OCT4* enhancer/promoter in 293T cells did result in *POU5F1* gene induction, suggesting that the function of chromatin loops is highly locus‐ and context‐dependent. Using CLOuD9, Wang et al. targeted two loci straddling the Dppa5a (Developmental Pluripotency‐Associated 5A) TAD boundary to drive TAD fusion.^[^ ^120^ ^]^ Looping was validated by 3C and Hi‐C, which showed increased inter‐TAD contacts indicative of TAD merging. Functionally, enforced linking upregulated Dppa5a early in reprogramming and boosted iPSC generation. Interestingly, complementary CRISPR boundary deletion produced similar TAD fusion and reprogramming gains.^[^ ^120^ ^]^

Unlike CLOuD9, which relies on ABA‐induced dimerization and requires two different dCas9 orthologs, LADL uses a single dSpyCas9–CIBN anchor targeting both genomic sites and a soluble CRY2 “bridge” that heterodimerizes with CIBN upon blue light exposure (Figure 2D).^[^ ^121^, ^124^ ^]^ This approach enabled rapid and reversible DNA looping, mediating *de novo* long‐range contacts spanning hundreds kb in sequence between the *Klf4* enhancer and the *Zfp462* promoter. Functionally, the LADL‐mediated looping resulted in a (modest) activation of Zfp462 expression and the underlying loop strength, as determined by 5C, was sensitive to illumination parameters and locus context.

While undoubtedly powerful in the context of their demonstrated use cases, the inducible dimerization systems above do not allow multiplexing, i.e., establishing selective interactions between multiple locus‐pairs. The reason is that using multiple copies of dCas9‐Zips, CLOuD9, or LADL would lead to stochastic or random loop formation, rather than predictable, locus‐specific interactions. Conceptually, overcoming this limitation requires pairwise‐specific and orthogonal linking mechanisms so each intended locus pair can be induced without cross‐talk.

Qin et al. developed the Bioorthogonal Programmable Chromatin Looping (BPCL) platform, which differs from CLOuD9 and LADL by using orthogonal “click” chemistries to link dCas9/sgRNA complexes rather than reversible protein dimerizers.^[^ ^122^, ^125^ ^]^ BPCL encodes adaptor‐target sites into engineered sgRNA scaffolds and delivers complementary single‐stranded clickable oligonucleotide adaptors (ssCOAs; SPAAC: azide–DBCO; SPIEDAC: tetrazine–TCO) that ligate in situ to juxtapose targeted loci with rapid kinetics and minimal cross‐talk (Figure 2E).^[^ ^122^ ^]^ Exploiting two orthogonal chemistries, the authors induced independent *POU5F1* and *SOX2* promoter–enhancer loops in the same 293T cell and thereby activated endogenous transcription. Moreover, a multi‐input BPCL variant was designed to force the co‐juxtaposition of three *POU5F1* elements (promoter, distal 5′ enhancer, and 3′ enhancer); simultaneous provision of SPAAC‐ and SPIEDAC‐ssCOAs produced stronger locus‐wide cross‐linking as well as a larger increase in *POU5F1* RNA than single‐loop induction, indicating that higher‐order, multiway hubs can potentiate transcription beyond pairwise looping. Remarkably, these effects were reversible via UV light, since oligonucleotide adaptors contained 365 nm light sensitive groups, or oligo removal.

While BPCL uses exogenous, orthogonal chemistries to build multi‐input, photocleavable hubs, a conceptually minimalist alternative was recently introduced by Yang et al.:^[^ ^123^ ^]^ a double‐guide RNA (dgRNA) in which two sgRNA units are covalently linked on a single sugar‐phosphate backbone so that one RNA molecule can simultaneously recruit two d*Spy*Cas9 molecules to distinct genomic sites (Figure 2F). In *E. coli*, the authors showed that dgRNAs can mimic a LacI‐mediated loop to repress *lacZ*, with loop formation evidenced by atomic‐force microscopy and functional gene repression assays. The dgRNA design is compact and requires only one dgRNA per loop to be formed, is easily programmable by spacer sequence swapping, and thus particularly attractive for multiplexing and for use with size‐constrained delivery vectors. However, to date the dgRNA‐mediated looping approach has only been demonstrated in the *E. coli* prokaryote and lacks intrinsic inducibility or reversibility. It will be interesting to see if this approach is also compatible with the mammalian chromatin context and if it can be paired with switchable dCas9‐ or anti‐CRISPR systems^[^ ^126^, ^127^, ^128^, ^129^, ^130^ ^]^ to enable temporal control over loop formation.

### 4.3 Choosing from the Menu of Available *De Novo* Looping Strategies

**Table** **1** summarizes the key advantages as well as technical trade‐offs of current DNA‐looping platforms, which all follow the same basic logic: a customizable, sequence‐specific DNA binder (ZFP, TALE, or dCas9) localizes an interaction module to defined loci and loops are established either by protein dimerizers, engineered RNA scaffolds, or bio‐orthogonal chemistry. Systems that use two orthogonal dCas9 fusions (dCas9‐Zips; CLOuD9) have been demonstrated in bacterial and mammalian cells, but they require co‐delivery/‐expression of two large proteins and cannot be cleanly multiplexed.^[^ ^115^, ^118^ ^]^ LADL reduces protein complexity by using a single dCas9 anchor with CRY2–CIBN photodimerization and affords fast temporal control.^[^ ^121^ ^]^ By contrast, BPCL implements orthogonal click chemistries to link dCas9 complexes and thereby enables selective, simultaneous formation of multiple, higher‐order hubs; it is also reversible, although the requirement of using toxic UV light for photocleavage may cause unpredictable side‐effects. Moreover, BPCL requires exogenously supplied, modified oligonucleotide adaptors.^[^ ^122^ ^]^ The dgRNA approach is conceptually the most compact and, on top, fully genetically encoded. In this approach, one covalently linked double‐guide RNA recruits two identical dCas9 proteins, making it attractive for multiplexing and size‐constrained delivery, but it has so far only been validated in prokaryotes.^[^ ^123^ ^]^ Protein‐only strategies based on ZFP and TALE are fully genetically encoded as well, and informative as proofs of principle. However, they are laborious to design and scale.^[^ ^36^, ^77^ ^]^ Taken together, choosing from the menu of available tools should be informed by experimental priorities, such as the need for temporal reversibility (CLOuD9, LADL, BPCL), selective multiplexing (BPCL), or minimal payload (dgRNA, pending mammalian validation). After selecting a platform, researchers should rigorously validate loop formation, pairing specificity, and transcriptional effects in their chosen cell type and experimental conditions, since delivery, expression level, cell, and chromatin context will profoundly influence both tool performance as well as regulatory outcomes. When paired with orthogonal readouts, e.g., 3C/5C/Hi‐C, imaging, and transcriptomics, these engineered‐looping tools provide tractable perturbations that can be used to test whether specific contacts causally affect gene expression rather than merely correlate with it.

**Table 1:** Programmable DNA‐looping tools, technical overview.

	Components co‐delivered/‐expressed	Fully genetically encodable	Inducible	Reversible	Loci tested	Cell lines /Organism tested	Readout	Distance looped	Refs.
ZFP‐SA^a)^	2 proteins	Yes	No	No	*HBB*	c, K562	RT‐qPCR, 3C	up to 40 kb	[77]
TALE‐SA^a)^	2 proteins	Yes	No	No	*SHH*	mESC	RT‐qPCR	up to 450 kb	[36]
dCas9‐Zips	2 proteins 2 sgRNAs	Yes	No^c)^	No^c)^	*lacI*	*E. coli*	3C, LacZ assay	up to 11.7 kb	[115]
CLOuD9	2 proteins 2 sgRNAs	Yes	Yes, chemically inducible with ABA	Yes, by washout of ABA	*HBB, POU5F1*	HEK293T, K562	3C, RT‐qPCR, Hi‐C	up to 167 kb	[118, 120]
LADL	2 proteins 2 sgRNAs	Yes	Yes, inducible with blue light	Yes, by stopping illumination	*Klf4*, *Zfp462*	mESC	5C, RT‐qPCR	up to 540 kb	[121]
BPCL^b)^	1 protein 2 sgRNAs 2 DNA oligos	No	No^c)^	Yes, via UV light	*POU5F1*, *SOX2*	HEK293T	3C, RT‐qPCR	up to 14.8 kb	[122]
dgRNA^b)^	1 protein 1 dgRNA	Yes	No^c)^	No^c)^	*lacI*	*E. coli*	LacZ assay	up to 4.5 kb	[123]

^a)^ requires considerable engineering to adapt to new loci;

^b)^ Multiple loci can be targeted in the same cell;

^c)^ Combining these strategies with switchable dCas systems may allow for inducibility and/or reversibility, but this is yet to be demonstrated.

### 4.4 Disruption of Chromatin Interactions

While forcing enhancer–promoter interactions can provide insight into their functional importance, this approach has some limitations. Primarily, it requires the absence of this particular contact in the first place. Activation or transcriptional upregulation of these genes via a loop may require additional effectors, not allowing to distinguish regulatory contacts that are necessary but not sufficient for gene activation from dispensable contacts. In line with this, forcing an enhancer–promoter contact does not necessarily result in gene activation.^[^ ^118^ ^]^ Contact perturbations at active loci would avoid the requirement for additional activators and could therefore complement technologies for forced looping.

Inducible protein degradation systems have been used for rapid depletion of essential proteins allowing to disrupt enhancer–promoter interactions *en masse* by removing structural parts of chromatin loops. Examples include cohesin and its regulatory components,^[^ ^21^, ^69^, ^70^ ^]^ CTCF^[^ ^131^ ^]^ Mediator^[^ ^35^, ^85^, ^86^ ^]^ LDB1,^[^ ^78^ ^]^ PRC1^[^ ^69^ ^]^ and YY1.^[^ ^76^ ^]^ While this approach is powerful, it is difficult to distinguish global transcriptional effects from locus‐specific effects mediated strictly through changes in local chromatin topology.

Another approach to disrupt interactions involves manipulations of CTCF binding sites that demarcate TAD boundaries. The deletion or inversion of CTCF binding sites can reduce the frequency of interactions between promoters and enhancers due to the disruption of the TADs in which they reside. This was achieved at the *HOXA* locus using zinc finger nucleases (ZFNs)^[^ ^107^ ^]^ and at the *Malt1*, *Sox2*, and *Fbn2* loci with CRISPR‐Cas9.^[^ ^101^ ^]^ In a similar manner, TALEs with nuclease activity (TALENs)^[^ ^132^, ^133^ ^]^ were used to delete the DXZ4 microsatellite, which is rich in CTCF motifs, thereby disrupting the higher‐order structure of inactive X chromosome.^[^ ^106^ ^]^ Insertions of CTCF binding sites have also been used to prevent chromatin contacts through loop‐extrusion dependent insulation.^[^ ^134^, ^135^ ^]^ Finally, Benabdallah et al.^[^ ^36^ ^]^ recruited TALEs fused to proteins exhibiting chromatin unfolding activity to the *Shh* locus leading to gene activation.

These approaches are powerful in their disruption of interactions, but the need for targeted genomic integrations or complicated protein engineering limit scalability. Moreover, most loop disruption approaches depend on affecting endogenous proteins, the functional consequence of which can be manifold.

Taken together, contact perturbations allow for a better understanding of their relevance for active transcription. In the future, more specific and easily adaptable disruption methods need to be developed to enable targeted manipulations at a larger scale.

## 5 Conclusion

Programmable looping is advancing from proof of principle to an actionable perturbation platform for testing whether particular chromatin contacts drive gene expression and cell behavior rather than merely correlate with them. Prominent examples include CLOuD9, which uses abscisic‐acid gated PYL1–ABI1 dimerization on orthogonal dCas9s,^[^ ^118^ ^]^ LADL, which exploits blue‐light CRY2–CIBN photodimerization on a single dCas9 anchor,^[^ ^121^ ^]^ BPCL, which uses bioorthogonal click chemistry and sgRNA‐tethered adaptors to assemble selective, multiplexed hubs,^[^ ^122^ ^]^ and dgRNA, a compact double‐guide design recently demonstrated in bacteria.^[^ ^123^ ^]^

The primary barriers to further development and large‐scale deployment of these tools are practical rather than conceptual. The most important issues are efficiency, i.e., the ability to induce loops reliably and robustly enough to probe measurable regulatory outputs across diverse contexts; scalability, meaning multiplexing and delivery, which is currently hampered by trade‐offs between versatility, e.g., dynamic control via inducers, and compactness of available tools; and specificity, meaning cross‐talk free pairing. Three routes appear especially promising to address these constraints.

One route is exploring compact, genetically encodable architectures. In the context of CRISPR‐based strategies, double‐guide RNAs minimize payload by encoding two guides on one backbone and by requiring only a single Cas9 variant to be present in the cell. In principle, the dgRNA approach could also be paired with inducible CRISPR control such as light‐ or drug‐switchable anti‐CRISPRs or dCas9s to restore temporal gating without additional protein fusions.^[^ ^123^, ^126^, ^127^, ^128^, ^129^ ^]^ In parallel, shifting from Cas9 to smaller CRISPR effector proteins may ease expression and delivery, mitigate metabolic burden and thus allow denser multiplexing. In this context, compact CRISPR effectors such as CasX/Cas12e, CasΦ/Cas12j, and engineered Cas12f variants offer potentially attractive chassis for loop‐writing platforms that fit within single AAVs and leave room for regulatory add‐ons.^[^ ^136^, ^137^, ^138^ ^]^ Their suitability in the context of bivalent binders for *de novo* chromatin looping, however, still needs to be explored.

A second promising route would be leveraging naturally evolved bivalent RNA‐based systems, such as the recently discovered bridge RNAs, and re‐engineering them toward new classes of synthetic bispecific chromatin tethers.^[^ ^139^, ^140^ ^]^

A third, longer‐term route is the *de novo* design of bi‐ or multivalent DNA‐binding proteins. Rapid advances in AI‐guided protein engineering have already produced compact, programmable proteins with sequence‐selective DNA recognition^[^ ^141^ ^]^ providing a realistic stepping stone toward fully synthetic chromatin looping agents.

Beyond improving looping tools themselves, a major challenge is the lack of standardized benchmarks and simple strategies for comparing their efficacy and robustness. New methods in this area are typically validated and tuned within a single experimental context (cell type, expression level, timing, readout), which prevents objective cross‐platform assessment. Future work should therefore establish shared benchmark assays and quantitative metrics to identify critical design parameters and feed them back into the tool engineering cycle.

We are optimistic that exploring these routes will eventually yield optimized tools to enable new mechanistic experiments that are difficult or impossible today. For example, they will enable direct tests of DNA looping necessity and sufficiency at endogenous loci. Imposing or disrupting a single contact and asking whether that change is necessary or sufficient for altering transcription will facilitate discriminating causal relationships from merely correlative observations. Moreover, systematically varying anchor spacing or induction duration could provide quantitative insights into the minimal proximity and dwell time required to trigger certain regulatory outcomes. Multiplexed perturbations would further allow systematic addition or removal of individual enhancers from multi‐enhancer hubs, revealing whether elements act additively, synergistically, or redundantly. On top, the rapid, time‐gated loop induction paired with high‐temporal‐resolution readouts (live imaging or nascent‐RNA assays) may support establishing the order of events and thus resolve causal direction and orchestration of dynamic and complex chromatin topologies. Crucially, pairing engineered proximity with orthogonal readouts for physical distance and dynamics or transcriptional output, such as 3C/Hi‐C, DNA‐FISH, or live two‐locus imaging and single‐cell nascent transcriptomics, will allow the field to parse which contacts are drivers and which are passengers of regulatory programs.

Apart from techniques for direct looping via tethering of precisely defined loci, various strategies have been developed to reposition chromatin to nuclear subcompartments (lamina, nucleolus, PML bodies/speckles) or to engineered condensates. While these do not enforce a specific A‐B loop, they can co‐concentrate targeted loci within the same small volume, thereby increasing encounter frequencies and modulating looping outcomes. Early operator–tethering fused LacI to inner–nuclear‐membrane proteins to pull lacO‐tagged loci to the nuclear periphery.^[^ ^142^ ^]^ CRISPR‐based platforms broaden this idea: CRISPR‐GO uses dCas9 adaptors to move endogenous loci to the periphery, nucleolus, PML bodies, or speckles in an inducible and reversible manner.^[^ ^143^ ^]^ In yeast, CRISPR‐PIN anchors dCas9‐marked loci to defined perinuclear landmarks to control radial positioning.^[^ ^144^ ^]^ Orthogonally, optogenetic condensate builders such as CasDrop nucleate phase‐separated hubs at targeted sites, clustering nearby chromatin and effectively raising local contact probabilities.^[^ ^145^ ^]^ In loop‐engineering workflows, these repositioning tools could provide complementary control: they can potentiate engineered loops by co‐recruiting partners into one hub or stress‐test loop function in repressive contexts. Their main limitation is specificity: co‐recruited loci share a compartment but may not engage each other, so direct pairwise tethers will remain essential when defined loops are required.

Together, these rapidly arising engineering capabilities have clear translational implications, because disruption of 3D genome organization contributes to various human diseases. Structural rearrangements that cause enhancer hijacking or TAD boundary disruption can aberrantly activate oncogenes, as exemplified by GFI1 (growth factor independent 1) enhancer rewiring in medulloblastoma.^[^ ^29^ ^]^ Developmental disorders such as limb malformations and neurodevelopmental syndromes have been traced to TAD boundary deletions and enhancer miswiring, as shown for Eph receptor A4 (EPHA4)‐associated limb phenotypes and for 16p11.2 copy‐number alterations.^[^ ^26^, ^146^ ^]^ In this context, programmable looping offers a disease‐modeling dial to recreate suspected pathogenic contacts, to test whether they are sufficient for misregulation, and to reverse‐engineer topology to probe rescue. In the longer term, compact, orthogonal, and inducible loop‐writing systems could even complement sequence editing in therapeutic strategies that modulate expression without altering coding sequence, for example, where rerouting enhancer connectivity yields clinically meaningful transcriptional changes such as increased fetal hemoglobin.

Finally, provided we can overcome the remaining substantial technical hurdles, we envision that the loop engineering approaches reviewed here could be repurposed as architectural “hardware” to program chromatin organization entirely *de novo*. By enabling formation, disruption, and rewiring of specific DNA loops, long‐range chromatin contexts, and the spatial positioning of loci, these tools may ultimately allow researchers to construct bespoke genome architectures in living cells – or even in bottom‐up synthetic cell entities – opening routes to precisely engineered chromatin states on demand.

In sum, bringing molecular engineering into the chromatin‐conformation field is poised to do more than map who is near whom: it will enable programming who meets whom, when they meet and for how long. Thereby we will not only be able to decisively distinguish causation from correlation but ultimately route gene expression and cell state through rational chromatin‐topology design.

## Conflict of Interest

The authors declare no conflict of interest.
